# Using Wearable Video Cameras to Assess Screen Use Contexts in Preschool-Aged Children: Pilot Observational Study

**DOI:** 10.2196/85215

**Published:** 2026-02-26

**Authors:** Amanda Machell, Katherine Downing, Simone J J M Verswijveren, Kylie D Hesketh

**Affiliations:** 1 Institute for Physical Activity and Nutrition (IPAN), Deakin University, 1 Gheringhap Street, Geelong, 3220, Australia, 61 392446613

**Keywords:** early childhood, screen time, smartphone, television, tablet

## Abstract

**Background:**

Wearable video cameras may offer a feasible approach to assess the contexts of screen use (eg, screen content and co-use) among preschool-aged children.

**Objective:**

The objective of this study was to assess the contexts of screen use among preschool-aged children using wearable video cameras.

**Methods:**

Children aged 2 to 5 years from Melbourne, Australia, wore a video camera for 1 day in the home environment during May 2023. One researcher manually coded video footage second by second; 15% was double coded for reliability. Coding included device type, screen activity, screen content classified using Common Sense Media ratings, streaming service, setting, social interaction, screen multitasking, and concurrent behaviors.

**Results:**

A total of 37,944 seconds (10.5 hours) of video camera footage from 8 children were identified and coded as screen use, equating to 21.8% (37,944/174,290) of total camera wear time (range 0.3%-74.0%). Screen use was predominately characterized by program viewing (n=37,461, 98.7% seconds) on televisions (n=34,192, 90.1% seconds) in the lounge room (n=33,710, 88.8% seconds). Programs scored low for educational value (mean 1.7, SD 1.4 of 5 stars), and approximately one-third (3/9, 33.3%) of programs were classified as appropriate for an age older than that of the children in this sample. Screen multitasking was rare (n=46, 0.1% of seconds), and coviewing occurred in approximately one-third of all screen use (n=11,010, 29%).

**Conclusions:**

Contexts considered beneficial for development (eg, educational and age-appropriate content) were infrequently observed. This suggests that interventions to equip parents with practical strategies to identify genuine educational content and recognize and avoid age-inappropriate content are warranted. However, our small sample size limits generalizability.

## Introduction

Young children are the fastest-growing users of screens [1], with 2025 estimates indicating that, by the age of 6 years, 62% of children own a tablet, and 10% own a smartphone [2]. The widespread availability of screen-based devices has fueled an ever-growing debate regarding their potential impact on preschool-aged children’s health and development. As such, guidelines from the World Health Organization and several individual countries focus on setting time limits, recommending that children aged 2 to 5 years have no more than 1 hour of screen time per day [3-5]. There is emerging evidence suggesting that the context in which screen use occurs may alter associations with health and developmental outcomes. For example, an umbrella review of 102 meta-analyses (youth aged 0-18 years) indicated that coviewing (eg, with a parent) and educational screen content were associated with better literacy outcomes [6]. Additionally, results from a meta-analysis of 100 studies (children aged <6 years) indicated that age-inappropriate content was associated with worse psychosocial outcomes, whereas co-use was associated with better cognitive outcomes [7]. As such, in addition to time limits, pediatric societies of the United States, the United Kingdom, and Canada emphasize quality contexts, recommending that parents coview with their children, choose high-quality programming (eg, educational content), and avoid violent content [8-10].

Much of the evidence informing screen guidelines and recommendations is limited by methodological factors [11,12]. Evidence on preschool-aged children’s screen use relies largely on parent-reported measures such as questionnaires and time use diaries, which are prone to bias (eg, social desirability and recall bias) [13]. Additionally, these measures typically focus on traditional media (ie, television and computer and video games) while overlooking newer devices (ie, tablets and smartphones) or aggregate total daily screen use [11,12,14]. Such methodology may fail to capture a comprehensive understanding of the nuanced contexts in which preschool-aged children engage with screens, such as content consumed, screen activity (eg, program viewing and communication), social interaction (ie, coviewing vs solo viewing), and concurrent behaviors (eg, eating and playing). Thus, measures capable of providing a nuanced understanding of the context of screen use among preschool-aged children are needed to better understand impacts on health and development and better inform guidelines. Wearable video cameras may provide a promising solution as they can continuously capture when, where, and how children interact with screens, providing rich contextual information on screen use.

To our knowledge, only 1 study has used wearable *video* cameras to examine screen use, albeit in an older age group [15]. That study recorded 1081 hours of video footage during the 2 hours before sleep among adolescents (N=83; aged 11-14 years) in New Zealand [15]. Results indicated that most participants used smartphones (n=72 participants; 87%) and televisions (n=70 participants; 84%) during the presleep period, but multiscreen use was also common (n=63 participants; 76%) [15]. The most common screen activities that adolescents engaged in before bed were program viewing (n=76 participants; 92%), browsing (n=68 participants; 82%), communication (n=65 participants; 78%), gaming (n=59 participants; 71%), and social media use (n=51 participants; 62%) [15].

Other studies have used wearable *photo* cameras to examine screen use among adolescents in New Zealand (N=108; aged 11-13 years; n=120,708 images) [16] and Australia (N=10; aged 13-17 years; n=71,396 images) [17]. Results from both studies indicated that television was the most used device (42% of screen time; 36% of images), followed by computers (32% of screen time; 32% of images), mobile devices (13% of screen time; 29% of images), and tablets (13% of screen time; 4% of images) [16,17]. The most common screen activity was program viewing (27% of screen time), followed by games (24% of screen time), social activities (eg, social media and SMS text messaging; 8% of screen time), and using the internet (including online shopping and watching videos on YouTube; 7% of screen time) [16]. Thomas et al [17] reported screen activity separately for each device and further classified screen use in terms of content, setting, social interaction, and concurrent behaviors. Televisions were used primarily for gaming (54% of images), whereas smartphones and tablets were mostly used for program viewing (50%-51% of images). Most screen content was classified as recreational (70% of images), was consumed in the living room (58%) or bedroom (30%), and involved limited co-use with adults (3%) or children (2%), and there were few instances of concurrent behaviors (eg, writing using a pen and paper or having a snack or meal; 13% of images) [17].

While those studies offer initial insights into the potential for wearable cameras to capture screen use contexts, several key evidence gaps remain. First, all prior investigations have focused exclusively on adolescents [15-17], which is unlikely to generalize to preschool-aged children. For example, survey data show that preschool-aged children’s daily screen use is typically dominated by television viewing, with little engagement in gaming, social media, or video chatting [2]. In contrast, adolescents’ screen use is typically more heavily dominated by social media, gaming, using the internet, and video chatting [18]. Second, most (2 out of 3) prior camera studies have considered a limited set of screen use contexts (ie, device type and screen activity) [15,16]. Other contextual factors such as social interaction and screen content are important to explore due to their potential impact on early childhood development [7]. Finally, with the exception of 1, prior studies have relied on wearable photo cameras rather than video-based devices [16,17]. Photo cameras typically capture images at fixed intervals (eg, every 10 seconds), which may result in missed instances of screen use. In contrast, wearable video cameras provide continuous recording, enabling a more comprehensive understanding of children’s screen use contexts.

Extending wearable camera research to early childhood offers an opportunity to capture the nuanced environmental and social contexts of screen use, which are difficult to measure using traditional self-report approaches. To address these limitations, this study aimed to explore a broad range of screen use contexts among preschool-aged children using wearable video cameras. The screen use contexts considered included device type, screen activity, screen content, streaming service, setting, social interaction, coviewing partners, multiscreen activity, and concurrent behaviors.

## Methods

### Ethical Considerations

Ethics approval was obtained from the Deakin University Human Research Ethics Committee (HEAG-H 04_2023). Written informed parental consent and verbal child assent were obtained before data collection. Household members were asked to provide verbal permission. Data were collected during May 2023 and June 2023 and analyzed between April 2025 and June 2025. Data collected are stored on a restricted shared drive at Deakin University, are presented in aggregate form, and no video footage is published, to protect the identity of participants. Participants received an Aus $10 (US $7.08) supermarket voucher per child participating in the study as a token of appreciation for their time and participation.

### Study Design

Data for this pilot observational study were drawn from a larger study that aimed to examine children’s physical activity and sedentary behavior in home environments using a combination of wearable devices. Participants wore 3 movement sensors (activPAL [PAL Technologies Ltd], ActiGraph GT3X+, and ActiGraph GT9X Link) and a wearable video camera (Vimel ultrahigh-definition body camera; 1296p; 36 MP) during waking hours. While all devices contributed to the broader assessment of children’s movement behaviors, this pilot study focused specifically on the screen use data captured via the wearable video cameras.

### Participants and Recruitment

Participants included parents and caregivers (hereafter referred to as “parents”) and their children aged 2 to 5 years from Melbourne, Victoria, Australia. To be eligible, parents needed to be able to read English and be aged >18 years. Recruitment took place through convenience and snowball sampling, with participants identified through the research team members’ (KD, KDH, and SJJMV) known networks; institute-wide emails; and a public post on the institute Facebook page (October 30, 2024), which was also shared with the research team’s networks. Potential participants were emailed a link to the online study recruitment page (via Qualtrics [Qualtrics International Inc]), where they could view and download an information sheet, undergo eligibility screening, and provide consent should they agree to take part in the study. During the consent process, parents were asked to report their children’s date of birth; sex; and residential address, including postcode. Socioeconomic status was classified using the Australian Bureau of Statistics Index of Relative Socioeconomic Advantage and Disadvantage, which ranks postcodes in Australia according to neighborhood socioeconomic conditions from 1 (most disadvantaged) to 10 (most advantaged) [19].

### Measures

#### Wearable Video Cameras

Parents were asked to attach a wearable video camera (Vimel ultrahigh-definition body camera; 1296p; 36 MP) to the front of their children’s clothing using a clip and a quick-release lanyard for additional safety. The camera had a size of 75 × 57 × 30 mm and a battery life of approximately 8.5 hours, captured a 140-degree field of view, and was set up to record video only (no audio) for privacy reasons. The cameras were posted to parents along with detailed instructions on how to wear, charge, and turn off the camera (eg, when going to the bathroom or getting undressed). Children were asked to wear the camera exclusively in the home environment for a total of 8 to 10 hours, which could be spread over multiple days. Parents were given the opportunity to view (and delete if necessary) video footage before it was viewed by the research team. The research team removed any accidentally captured video data that were considered a breach of privacy (eg, diaper changes). Video data were date- and time-stamped and permanently deleted from the cameras following download and storage on a restricted share drive.

#### Image Coding

Video footage data for each participant were manually coded (second by second) by a research assistant using a 2-stage manual coding process in Microsoft Excel. First, all video data were coded to identify the activity undertaken (eg, screen based, reading, playing, eating, physical activity, and socializing). Second, video data that were initially coded as screen based were further classified using a comprehensive coding protocol adapted from similar studies [16,17] (Multimedia Appendix 1). Video footage data were coded for device type, screen activity, screen content, streaming service, setting, social interaction, coviewing partners, multiscreen activity, and concurrent behaviors. Screen content was classified using Common Sense Media’s ratings for quality (ie, positive messaging, positive role models, violence and scariness [reverse scored], and educational value) and age-appropriateness [20]. Consistent with previous studies [21], each quality indicator was rated on a scale from 0 to 5, where 0 was “not present” or “not applicable,” 3 indicated a fair amount of that content, and 5 indicated a lot of that content.

Footage that was “blocked” or had an obscured view was considered inconclusive for determining screen use behavior, especially if the camera angle was directed away from the screen or the footage was blackened due to obstruction. Blocked or obscured footage was partially coded. For example, if a device type was known to be in use but other screen information was blocked, the device type, but no other screen use information, was coded. A subset of the identified screen time video footage (15%) was double coded by the first author (AM).

### Data Analyses

Analyses were conducted in Stata (version 18.0; StataCorp LLC). Camera wear time was calculated as the total number of minutes during which video footage was recorded. Descriptive statistics (means and proportions) were used to describe the sample and calculate time spent in each screen use context. Independent-sample 2-tailed *t* tests were performed to examine differences between mothers and fathers in terms of coviewing session duration and total coviewing duration. Interrater reliability for the double-coded footage was calculated using the κ statistic and interpreted as follows: κ=0.00 to 0.20 indicated slight agreement, κ=0.21 to 0.40 indicated fair agreement, κ=0.41 to 0.60 indicated moderate agreement, κ=0.61 to 0.80 indicated substantial agreement, and κ=0.81 to 1.00 indicated almost perfect agreement [22].

## Results

### Interrater Reliability

Coder agreement was high across most screen use contexts. Perfect reliability was achieved for device type, multiscreen activity, and setting (κ=1.00), whereas screen content (κ=0.95), streaming service (κ=0.94), social interaction (κ=0.87), screen activity (κ=0.84), and coviewing partners (κ=0.82) exhibited very strong agreement. In the initial coding round, the concurrent behavior categories of play and fidgeting were differentiated using time-based criteria, whereby shorter durations were coded as “fidgeting” and longer durations were coded as “playing.” However, this interpretation was somewhat subjective, and the coding protocol was subsequently revised to distinguish between “object-based” and “non–object-based” play and fidgeting, which achieved substantial agreement (κ=0.64).

### Sample Characteristics

A total of 11 parents of preschool-aged children consented to take part in the study; however, 2 (18.2%) of the participants refused to wear the video camera. The final sample comprised 9 children, who recorded a total of 174,290 seconds (48.4 hours) of video camera footage over the observation period. Mean wear time was 5.4 (SD 2.9; range 0.3-10.1) hours. A total of 37,944 seconds (10.5 hours) of video camera footage from 8 children were coded as screen use, equating to 21.8% (37,944/174,290) of total camera wear time (range 0.3%-74.0%). Children were, on average, aged 3.8 (SD 1.1; range 2-5) years, 2 were girls, and 6 were boys. The child who recorded no screen use in their 0.3 hours (n=1245 seconds) of recording was a girl aged 3 years. Children were predominantly from advantaged backgrounds, with most residing in areas classified within Index of Relative Socioeconomic Advantage and Disadvantage deciles 8 to 10; 1 child was from an area classified as decile 6.

### Device Type, Screen Activity, Setting, and Social Interaction

Table 1 shows the duration of screen use by device type, screen activity, setting, and social interaction. Overall, screen use was quite homogeneous, with program viewing (37,461/37,944, 98.7% of seconds), televisions (34,192/37,944, 90.1% of seconds), and lounge room use (33,710/37,944, 88.8% of seconds) dominating screen activity, device type, and setting, respectively. Program viewing was the sole screen activity when using televisions (34,192/34,192, 100% of seconds) and tablets (2577/2593, 99.4% of seconds). In contrast, screen activity during smartphone use was more heterogeneous, including program viewing (692/1159, 59.7% of seconds), communication (345/1159, 29.8% of seconds), and browsing (122/1159, 10.5% of seconds). However, smartphone use accounted for only 3.1% (1159/37,944) of total screen use. Coviewing occurred in approximately one-third (11,010/37,944, 29% of seconds) of all screen use but differed by device type (television: 8053/34,192, 23.6% of seconds; smartphone: 567/1159, 48.9% of seconds; tablet: 2390/2593, 92.2% of seconds). Concurrent behaviors occurred during 38.6% (14,632/37,944) of screen use time, with eating snacks (5751/37,944, 15.2% of seconds) and non–object-based fidgeting (5847/37,944, 15.4% of seconds) being the most common, and these were relatively consistent across device types.

**Table 1.** Duration of screen use by device type, screen activity, setting, and social interaction.

	Smartphone (n=1159 seconds)	Television (n=34,192 seconds)	Tablet (n=2593 seconds)	Total (n=37,944)
Screen activity (seconds), n (%)				
Browsing	122 (10.5)	0 (0.0)	16 (0.6)	138 (0.4)
Communication	345 (29.8)	0 (0.0)	0 (0.0)	345 (0.9)
Program viewing^a^	692 (59.7)	34,192 (100.0)	2577 (99.4)	37,461 (98.7)
Setting (seconds), n (%)				
Child’s bedroom	43 (3.7)	0 (0.0)	0 (0.0)	43 (0.1)
Kitchen	853 (73.6)	0 (0.0)	0 (0.0)	853 (2.2)
Lounge room	234 (20.2)	30,883 (90.3)	2593 (100.0)	33,710 (88.8)
Parents’ bedroom	21 (1.8)	1125 (3.3)	0 (0.0)	1146 (3.0)
Playroom	8 (0.7)	2184 (6.4)	0 (0.0)	2192 (5.8)
Social interaction (seconds), n (%)				
Inconclusive	0 (0.0)	756 (2.2)	192 (7.4)	948 (2.5)
Coviewing	567 (48.9)	8053 (23.6)	2390 (92.2)	11,010 (29.0)
None	592 (51.1)	25,383 (74.2)	11 (0.4)	25,986 (68.5)
Concurrent behaviors (seconds), n (%)				
Eating meals	0 (0.0)	357 (1.0)	0 (0.0)	357 (0.9)
Eating snacks	293 (25.3)	5317 (15.6)	141 (5.4)	5751 (15.2)
Fidgeting—non–object-based	66 (5.7)	4686 (13.7)	1085 (41.8)	5847 (15.4)
Fidgeting—object-based	30 (2.6)	1627 (4.8)	109 (4.2)	1767 (4.7)
Playing—non–object-based	0 (0.0)	327 (1.0)	0 (0.0)	327 (0.9)
Playing—object-based	0 (0.0)	585 (1.7)	11 (0.4)	596 (1.6)
None	770 (66.4)	21,293 (62.3)	1247 (48.1)	23,312 (61.4)

a Program viewing predominately comprised watching programs and/or movies (35,028/37,461, 93.5% of seconds), but content selection (1034/37,461, 2.8% of seconds), waiting for autoplay (247/37,461, 0.7% of seconds), and loading screen content (27/37,461, 0.1% of seconds) were also categorized as program viewing.

### Duration of Coviewing Sessions

A total of 44 coviewing sessions were captured. Among these 44 coviewing sessions, after excluding 3 (6.8%) involving both parents (to avoid double counting), 6 (13.6%) involving another child, and 2 (4.5%) where the coviewing partner could not be determined, there were 19 (43.2%) sessions involving fathers (with or without other family members) and 14 (31.8%) involving mothers (with or without other family members). Coviewing sessions involving fathers lasted, on average, 4.9 (SD 9.2; median 0.85, IQR 0.2-5.0; range 0.1-38.6) minutes, whereas sessions involving mothers lasted, on average, 1.1 (SD 1.2; median 0.75, IQR 0.3-1.5; range 0.2-4.1) minutes; however, this difference was not statistically significant either before (*t*_31_=–1.50; *P*=.14) or after (*t*_30_=–1.55; *P*=.13) removing 1 outlier involving a coviewing session with a father lasting 38.6 minutes.

### Coviewing Partners

Approximately one-third (11,010/37,944, 29%) of screen use involved coviewing. Table 2 shows the coviewing partners of preschool-aged children. Approximately two-thirds (7396/11,010, 67.2% of seconds) of coviewing instances involved at least one parent, mostly the father (5558/11,010, 50.5% of seconds) but occasionally both parents (882/11,010, 8% of seconds) or the mother (956/11,010, 8.7% of seconds). Coviewing with an older child was also common (3556/11,010, 32.3% of seconds). After excluding coviewing sessions involving both mothers and fathers to avoid double counting, fathers spent longer coviewing with their children (mean 1111.6, SD 923.07 seconds) compared with mothers (mean 159.33, SD 112.46 seconds), and this difference was statistically significant (*t*_9_=–2.5; *P*=.03). However, after removing the outlier described above, this difference did not remain significant (coviewing involving fathers: mean 648.4, SD 680.42 seconds; coviewing involving mothers: mean 159.33, SD 112.46 seconds; *t*_9_=–1.75; *P*=.11).

**Table 2.** Coviewing partners (by duration) of preschool-aged children (total duration=11,010 seconds).

Coviewing partner	Duration (seconds), n (%)
Unable to tell	58 (0.5)
Father	4807 (43.7)
Father and mother	882 (8.0)
Father and older child	6 (0.1)
Father and younger child	745 (6.8)
Mother	564 (5.1)
Mother and grandmother	46 (0.4)
Mother and older child	346 (3.4)
Older sibling	3556 (32.3)

### Multiscreen Activity

There was very little multiscreen activity (46/37,944, 0.1% of all screen use), with a single recorded instance. This instance occurred for a brief duration (46 seconds) and involved watching sports on television while simultaneously watching a song video on a smartphone.

### Streaming Services

Children viewed a range of content across various streaming services, including YouTube Kids (9/20, 45% of programs), Netflix (6/20, 30% of programs), SVT Barn (2/20, 10% of programs), Spotify (1/20, 5% of programs), ABC iview (1/20, 5% of programs), and Foxtel (1/20, 5% of programs). For 8.8% (3345/37,944) of seconds of video footage (6 occurrences involving 3 participants), the streaming service could not be determined due to the screen content being inconclusive.

### Content Classifications

Content classifications were available for 45% (9/20) of the programs, representing 61.8% (23,441/37,944) of screen use footage. Programs scored approximately 3.0 out of 5 stars for positive messaging (mean score 3.0, SD 1.00; range 1-4 stars) and positive role models (mean score 3.1, SD 1.17; range 1-5 stars) and approximately 1 star for violence and scariness (mean 1.3, SD 1.22; range 0-3 stars) and educational value (mean 1.7, SD 1.41; range 0-4 stars). Approximately one-third (3/9, 33.3%) of programs were classified as appropriate for an older age than those of the children in this sample (ie, for the ages of ≥6, ≥8, or ≥10 years).

### Content Classifications and Social Interaction

Table 3 shows the mean content classification scores for coviewing vs solo viewing. Mean content classification scores differed according to social interaction. Specifically, mean scores were lower for educational value and positive messaging and higher for violence and scariness and positive role models when programs were coviewed than when programs were solo viewed.

**Table 3.** Content classification and social interaction of preschool-aged children. Total viewing time was 7171 seconds for coviewing and 15,528 seconds for solo viewing.

Content classification	Coviewing (number of stars), mean (SD)	Solo viewing (number of stars), mean (SD)	*t* test (*df*)	*P* value
Educational value	1.3 (1.2)	2.7 (0.9)	–95.4 (22,697)	<.001
Violence and scariness	2.0 (1.2)	0.9 (0.6)	96.1 (22,697)	<.001
Positive messages	3.2 (0.8)	3.6 (0.5)	–48.6 (22,697)	<.001
Positive role models	3.6 (1.0)	3.22 (0.42)	–37.1 (22,697)	<.001

## Discussion

### Principal Findings

This study appears to be the first, worldwide, to objectively assess preschool-aged children’s free-living screen use contexts using wearable video cameras. Our analysis yielded 4 main findings. First, screen use contexts among preschool-aged children were relatively homogeneous. Second, screen content was generally classified as low in terms of educational value, and one-third of content (3/9, 33.3% programs) was considered age inappropriate. Third, coviewing occurred in approximately one-third of screen use (11,010/37,944, 29%). Fourth, wearable video cameras provided a feasible approach for assessing preschool-aged children’s screen use contexts, albeit with some caveats. While a large amount of screen use data were captured (n=37,944 seconds), our findings should be considered alongside limitations associated with our small convenience sample (N=8).

This study showed that screen use contexts among preschool-aged children were relatively homogeneous. In our sample, almost all screen use involved program viewing of streamed or on-demand content on televisions in the lounge room. There was very little smartphone use or engagement in other screen activities such as communication or browsing. While the lack of variability in screen use may reflect our small, homogeneous sample, all from middle to high socioeconomic backgrounds, our focus on screen use in the home environment may have also influenced the results. Greater engagement with mobile devices and a wider range of screen activities might have been observed had we captured screen use outside of the home environment. For example, during interviews in a previous study, mothers (n=26) of preschool-aged children (aged 2-4 years) from the United Kingdom reported that they often allowed their children to use mobile devices outside of the home for a range of screen activities due to their multifunctionality, including playing games and taking and looking at photos, as well as program viewing [23]. Nonetheless, our finding that the television remains the primary source of screen use (34,192/37,944, 90.1%, of screen use in our sample) for preschool-aged children is consistent with results from previous studies [2,24,25] and suggests that older evidence from before the emergence of mobile devices is likely still relevant for this age group.

We found that the programs that preschool-aged children viewed were generally classified as having little educational content, and one-third of the programs (3/9, 33.3%) were classified as age inappropriate according to Common Sense Media ratings. Similarly, previous studies have shown that most content viewed by preschool-aged children is for entertainment rather than educational purposes [24,25]. It is possible that this finding reflects the nature of children’s television programs, with a 2019 examination of 88 popular children’s television shows in the United States indicating that, overall, shows aimed at children aged 2 to 5 years contained little educational content, scoring a mean of 2.44 out of 5 stars based on Common Sense Media classifications [21]. This is of concern given that substantial evidence demonstrates that high-quality educational television, such as Sesame Street, may benefit children’s cognitive, literacy, and social outcomes [26-29] and age-inappropriate content can negatively impact psychosocial outcomes [7]. An alternative explanation for the low educational value of the screen content observed in this study may be that, although screen guidelines from pediatric societies emphasize high-quality programming [8-10], this messaging may not be reaching parents. Thus, interventions aiming to support healthier screen use behaviors in preschool-aged children might focus on equipping parents with practical strategies to identify genuine educational content and recognize and avoid content that may be age inappropriate. Efforts could also involve engaging with industry to formally and scientifically evaluate content before making educational claims [10].

Coviewing in our sample occurred in approximately one-third of screen use (11,010/37,944, 29%). In contrast, Thomas et al [17] reported that just 16% of screen use involved coviewing among adolescents, with <1% involving interactive coviewing (n=7 images involving a laptop computer). However, the authors did not describe how this was coded from the camera images, making it difficult to ascertain how comparable this finding is to our data. In our study, we could not distinguish between interactive coviewing (eg, where another person asks questions or explains content) and passive coviewing (eg, where another person is present but not interacting with the child). This distinction is important as research suggests that interactive rather than passive coviewing can enhance language and cognitive development (eg, attention and memory) [24,30,31]. While the inclusion of audio data alongside video footage could be used to assess the quality of interactions, this approach may present additional ethical and feasibility challenges given the sensitive nature of recording in home environments. Future work may consider the inclusion of ecological momentary assessment to capture interaction during coviewing.

Wearable *video* cameras offer a promising and feasible approach to assessing preschool-aged children’s screen use in real-world contexts. Using these devices, we were able to objectively quantify a range of nuanced contextual information on preschool-aged children’s screen use. While wearable cameras reduce recall bias and offer a richer, more detailed account of children’s interactions with screens compared with self-report measures, several caveats must be considered. The success of this approach relies heavily on both parent consent and children’s willingness to wear the device consistently. A total of 11 parents of preschool-aged children consented to take part in this study, and most children (9/11, 81.8%) were willing to wear the device (mean wear time 5.4 hours), indicating that our protocol was feasible. Additionally, establishing strong interrater reliability is essential to ensure the reliability and replicability of the results. Our coding protocol achieved a high level of interrater reliability, supporting the robustness of our findings. Lastly, the substantial volume of video data generated from wearable video cameras makes coding time and labor intensive, underscoring the need to account for these factors in the study planning phase. As such, while wearable video cameras offer valuable insights into preschool-aged children’s screen use, their implementation requires careful ethical, practical, and methodological considerations.

There are a number of study limitations that should be acknowledged. While a large amount of screen use data were captured (10.5 hours), the small convenience sample and relatively homogeneous demographic characteristics limit generalizability to the wider preschool population. As such, future studies using larger samples are needed to overcome this limitation. Moreover, video footage data captured screen use exclusively in the home environment. As screen use outside the home environment may yield different patterns, future wearable camera studies examining screen use contexts both inside and outside the home environment are warranted. A previous wearable camera study in adolescents recorded camera footage both inside and outside the home environment, including on public transport, public food outlets, and community venues, suggesting that this approach is feasible and acceptable [17].

### Conclusions

Among a small sample, we showed that preschool-aged children’s screen use is relatively homogeneous, predominately characterized by program viewing of streamed content on televisions in the lounge room. While there was some positive messaging and role models, the content viewed by children was generally considered of little educational value and at times age inappropriate. Coviewing occurred during approximately one-third of screen use and more often with fathers than mothers. We showed that wearable video cameras offer a feasible and acceptable approach to collect more accurate and detailed data on preschool-aged children’s screen use contexts compared with traditional self-report measures. This study provides some useful insights for intervention development. However, being the first study of its kind in this population, further research on preschool-aged children’s screen use contexts both inside and outside the home using wearable cameras in larger samples is warranted to confirm our findings.

## Supplementary material

10.2196/85215 Multimedia Appendix 1 Coding protocol.
