# Supplementary material for: Using Wearable Video Cameras to Assess Screen Use Contexts in Preschool-Aged Children: Pilot Observational Study
Source: JMIR Pediatr Parent. 2026 Feb 26;9:e85215. doi: 10.2196/85215 (PMC12945350; doi:10.2196/85215)
Supplement: Multimedia Appendix 1 [file pediatrics-v9-e85215-s001.docx]

**Supplementary file 1: Coding protocol**

| **Screen context** | **Response options** | **Definition** |
| --- | --- | --- |
| Device type | Television |  |
|  | Smartphone |  |
|  | Tablet |  |
| Screen activity | Program | Watching any form of TV show, movie or video, including YouTube videos, on any device. E.g. Bluey, Smurfs. |
|  | Communication | Screen activities that involve communicating with other people. E.g. video chat, phone calls. |
|  | Browsing | Browsing the internet or looking at photos on any device. E.g. looking at Tokyo Disney website, looking at photos. |
|  | Inconclusive | Footage where it was clear the child was engaging with a screen, but it was uncertain what activity was occurring on the screen: this situation mostly occurred due to an interference of light or camera pointing away from the screen. |
| Screen content | Name of show | Captures the name of the show/movie/video/game/app the child is viewing. This is a free text field. |
|  | Content selection | Captures when the child and/or parent is selecting a program/screen content |
|  | Inconclusive | Footage where the screen activity was clear but it was uncertain what the screen content was: this situation mostly occurred due to an interference of light or camera pointing away from the screen. E.g., can see the child is watching a program but it is unclear what the program is. |
|  | n/a | Use when screen activity = inconclusive |
| Streaming service | Name of streaming service | The streaming service used to view screen content. E.g. Netflix, ABC iView, YouTube Kids |
|  | Inconclusive | Use if streaming service cannot be determined |
|  | n/a | Use if streaming service is not relevant. E.g. video chat, taking photos |
| Setting | Lounge/family room | The space within the home where the screen activity took place |
|  | Child's bedroom |  |
|  | Parent's bedroom |  |
|  | Playroom |  |
|  | Kitchen |  |
| Social interaction | None | Use if the child is alone when engaging with the screen activity |
|  | Co-viewing | Use if the child is engaging with the screen activity with another child or adult |
|  | Inconclusive | Use if it's unclear whether co-viewing occurred |
| Co-viewing partner | Parent - mother |  |
|  | Parent - father |  |
|  | Grandmother |  |
|  | Grandfather |  |
|  | Child |  |
|  | Inconclusive | Use if can see co-viewing is occurring (e.g. get a glimpse of a shoulder of a person sitting next to the child) but it’s unclear who that person is. |
|  | n/a | Use if social interaction = "None" |
| Second co-viewing partner | Parent - mother |  |
|  | Parent - father |  |
|  | Grandmother |  |
|  | Grandfather |  |
|  | Child |  |
|  | Inconclusive | Use if can see co-viewing is occurring but it’s unclear whether a second person is present. |
|  | n/a | Use if social interaction = "None" or if there is no second co-viewing partner |
| Concurrent behaviours | Eating snack | Refers to other behaviours child does while simultaneously viewing screen content |
|  | Eating meal |  |
|  | Playing (object-based) | The child is intentionally / purposefully engaging with a toy or object (e.g. stacking blocks, pretend play with a doll) |
|  | Playing (non-object-based) | The child is actively engaged in playful movement or social interaction without using objects (e.g. jumping around with a sibling) |
|  | Fidgeting (object-based) | The child is handling an object in a non-purposeful / non-intentional or repetitive way (e.g. moving an object from one hand to the other) |
|  | Fidgeting (non-object-based) | The child can’t sit still but instead keeps making changes to their position or fidgeting with their hair, fingers or clothing |
|  | None |  |
| Multi-screen activity | Yes | Use if child is using more than one device, simultaneously. E.g., watching TV while playing a game on a tablet |
|  | No | Use if child is not using more than one device, simultaneously. |
| Positive messaging | 0 star | Content classifications (from Common Sense Media) based on name of show, game etc |
|  | 1 star |  |
|  | 2 stars |  |
|  | 3 stars |  |
|  | 4 stars |  |
|  | 5 stars |  |
|  | Not available | Use if content classification is not available on Common Sense Media |
| Positive role models | As above |  |
| Violence and scariness | As above |  |
| Educational value | As above |  |
| Age recommendation | Age | Age recommendation (from Common Sense Media) based on name of program. |
